# PRESENCE OF DYSPHAGIA IN A COHORT OF PERSONS LIVING WITH DEMENTIA: OUTCOMES FROM A MULTIDISCIPLINARY CLINIC

**DOI:** 10.1093/geroni/igad104.3532

**Published:** 2023-12-21

**Authors:** Joanne Yee, Danielle Brates, Meredith Mackowicz-Torres, Raele Robison, Brian Lewis, Lindsay Clark, Ellen Wanninger, Nicole Rogus-Pulia

**Affiliations:** William S. Middleton Memorial Veterans Hospital, Madison, Wisconsin, United States; William S. Middleton Memorial Veterans Hospital, Madison, Wisconsin, United States; University of Wisconsin Madison, School of Medicine and Public Health, Madison, Wisconsin, United States; University of Wisconsin Madison, School of Medicine and Public Health, Madison, Wisconsin, United States; William S. Middleton Memorial Veterans Hospital, Madison, Wisconsin, United States; William S. Middleton Memorial Veterans Hospital, Madison, Wisconsin, United States; William S. Middleton Memorial Veterans Hospital, Madison, Wisconsin, United States; University of Wisconsin Madison, School of Medicine and Public Health, Madison, Wisconsin, United States

## Abstract

The Cognitive Care Clinic (CCC) provides evaluation for Veterans living with dementia. Speech-language pathology (SLP) is incorporated to facilitate early identification and proactive management of dysphagia. This project sought to describe dysphagia frequency in persons living with dementia (PWD) in CCC. Multidisciplinary assessments led to consensus diagnosis of dementia subtype. Stage was determined using the Functional Assessment Staging Tool (FAST). Validated dysphagia screening tools included Eating Assessment Tool (EAT-10), water swallow test (WST), and Test of Mastication and Swallowing Solids (TOMASS). Penetration-Aspiration Scale (PAS) scores were determined following videofluoroscopic swallow studies (VFSS); scores were classified as “safe” (PAS< 3) or “unsafe” (PAS>=3). Descriptive statistics were calculated for dementia subtype, stage, and dysphagia measures. 78 Veterans were diagnosed with dementia between 2017 and 2023 (mean age=77 years; 5% female) and completed dysphagia screening. The majority had Alzheimer’s disease (43%) and were in the mild disease stage (61%). 37% failed the WST, suggesting impaired safety. 49% failed the TOMASS, indicating masticatory inefficiency. 26% scored 3 or higher on EAT-10, indicating increased dysphagia risk. 22 patients completed VFSS; 55% exhibited unsafe swallows. No significant differences in EAT-10 total scores were observed between those with safe and unsafe PAS scores. Nearly half of this cohort demonstrated signs of swallowing impairment based on clinical testing and/or VFSS. Additionally, EAT-10 scores were low overall with no differences based on dysphagia status on VFSS, which suggests underreporting of dysphagia. These findings highlight the necessity of SLPs in dementia care teams to support early identification and management of dysphagia.

